# Current research on circular RNAs and their potential clinical implications in breast cancer

**DOI:** 10.20892/j.issn.2095-3941.2020.0275

**Published:** 2021-08-15

**Authors:** Diya Liu, Lin Fang

**Affiliations:** 1 Department of Thyroid and Breast Diseases, Shanghai Tenth People’s Hospital, Shanghai 200070, China

**Keywords:** Circular RNA, breast cancer, biomarker

## Abstract

Breast cancer (BC) is one of the most common cancers and the leading causes of death among women worldwide, and its morbidity rate is growing. Discovery of novel biomarkers is necessary for early BC detection, treatment, and prognostication. Circular RNAs (circRNAs), a novel type of endogenous non-coding RNAs with covalently closed continuous loops, have been found to have a crucial role in tumorigenesis. Studies have demonstrated that circRNAs are aberrantly expressed in the tumor tissues and plasma of patients with BC, and they modulate gene expression affecting the proliferation, metastasis, and chemoresistance of BC by specifically binding and regulating the expression of microRNAs (miRNAs). Therefore, circRNAs can be used as novel potential diagnostic and prognostic markers, and therapeutic targets for BC. This article summarizes the properties, functions, and regulatory mechanisms of circRNAs, particularly current research on their association with BC proliferation, metastasis, and chemoresistance.

## Introduction

The 3 most common cancers in women are breast, lung, and colorectal cancers, accounting for 50% of all new diagnoses. Breast cancer (BC) alone accounts for 30% of cancers in women. The 5-year relative survival rate for BC is 90%. A total of 279,100 new BC cases and 42,690 BC deaths in 2020 were predicted to occur in the United States^1^. Female patients worldwide still have the highest BC morbidity and mortality, despite advanced treatment approaches such as surgery, endocrine therapy, and targeted therapy. The discovery of early diagnostic biomarkers and novel therapeutic targets is of great significance for more effective treatment of BC. CircRNAs, a novel type of endogenous non-coding RNAs with covalently closed continuous loops, were first discovered in RNA viruses and cells of higher plants by Sanger et al.^2^ in 1976. Hsu et al.^3^ and Arnberg et al.^4^ have also found similar circular transcripts in HeLa cells and Saccharomyces cerevisiae mitochondria, respectively, through electron microscopy. Although circular RNAs were discovered decades ago, they cannot be detected by molecular techniques relying on poly (A) enrichment, because circular RNAs do not have free 3′ and 5′ ends^5^. Furthermore, because of back-splicing, the mapping algorithms in early transcriptome analysis could not directly align the sequenced fragments to the genome, thus resulting in the inaccurate understanding that circular RNAs were by-products arising from splicing errors. In recent years, with the development of high-throughput RNA-sequencing (RNA-seq) technologies and bioinformatics, and the establishment of large online circRNA databases, analyses of circRNAs have deepened.

Salzman et al.^6^ first indicated that circular RNAs are circular transcripts produced by the reverse transcription of mRNA precursors, which are abundant in diverse human cell types. Hansen et al.^7^ subsequently found that circular RNAs regulate the growth and development of organisms by acting as sponges of microRNA (miRNA). Since then, circRNAs have begun to enter the public view. Increasing evidence demonstrates that circRNAs are closely associated with a variety of non-tumor diseases (such as myocardial infarction^8^, diabetes^9^, multiple sclerosis^10^, and neurodegenerative^11^ diseases), and tumors including gastric cancer^12^, colorectal cancer^13^, hepatocellular carcinoma^14^, and acute promyelocytic leukemia^15^. Many types of circRNAs are differentially expressed in BC^16^. This differential expression is significantly associated with aspects including BC cell proliferation, apoptosis, and drug resistance. Moreover, because circRNAs are highly conserved and stable, they might be promising candidate biomarkers or targets for treatment of BC.

## Properties of circRNAs

CircRNAs are novel long-chain non-coding closed circular RNAs formed by pre-messenger RNA (pre-mRNA) reverse cleavage, which lack terminal structures. CircRNAs are widespread in organisms, such as humans^6^, mice, fruit flies, fish, and nematodes,^17^ and are found in various biological materials, such as plasma, saliva, and exosomes^6^. The expression patterns of circRNAs are often cell type-specific or developmental stage-specific^17,18^. According to gene structure annotation information, circRNAs are mainly divided into the following 5 categories: exon-derived circular RNA (exon circRNA, EcRNA)^19,20^; exons formed by back-splicing of upstream and downstream exons and intron retention, constituting exon-intron circular RNA (EIciRNA)^21^; intron-derived circular RNA (circular intronic RNA, ciRNA)^22^; fusion gene-derived circular RNA (fusion-circRNA, f-circRNA)^15,23^; and read-through circular RNA (rt-circRNA), formed through the transcriptional read-through of polymerase II (Pol II)^24^. Exon-derived circRNA is mainly found in the cytoplasm, which contains the largest proportion, whereas ciRNA and ElciRNA are mainly distributed in the nucleus^22,25^. CircRNAs now mainly refer to EcRNAs composed of one or more exons. EcRNAs have 3 main formation mechanisms: lariat-driven circularization^22^, intron-paring-driven circularization^26^, and RNA binding protein (RBP) circularization^27^.

The circRNA expression profile is conserved among mammals^28^. Because circRNAs do not contain 3′ and 5′ ends, they are not sensitive to ribonucleases, such as exonuclease or ribonuclease R, and are therefore stably expressed in cells^29^. Enuka et al.^30^ have found that epidermal growth factor induction strongly affects the abundance of mRNA and miRNA expressed in human mammary epithelial cells, but the abundance of circular RNAs homologous to these linear RNAs show no significant changes. By measuring the half-lives of dozens of known circRNAs and homologous linear RNAs, the authors have found that the average half-life of circRNAs is more than twice that of linear RNAs. This finding illustrates the stability of circular RNA in cells.

## Formation and regulatory mechanisms of circRNAs

**Figure 1:**
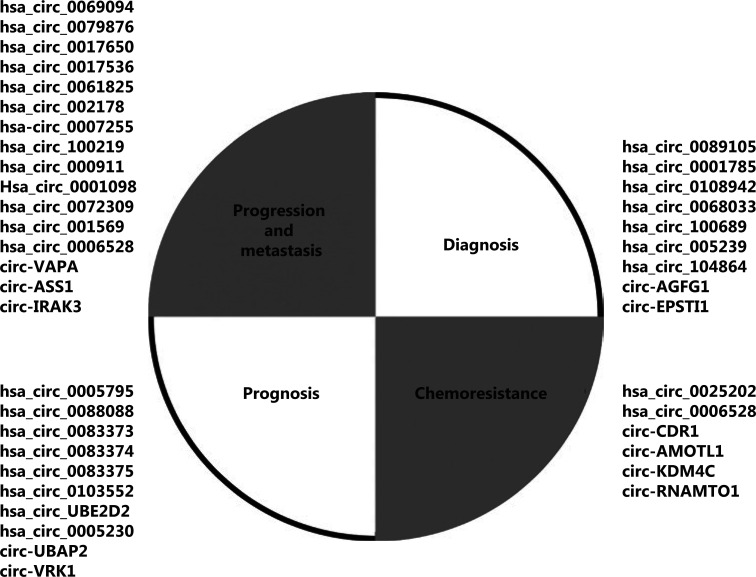
CircRNAs contribute to various aspects of breast cancer.

circRNAs are mainly generated by back-splicing of pre-mRNAs containing exons and introns. The circRNAs discussed in this article are EcRNAs. There are 3 main formation mechanisms of circRNAs (**Figure 1**). The pre-mRNA is half-folded so that non-adjacent exons are close to each other, and the intervening exons are spliced, forming a lasso intermediate. The lasso intermediate is spliced, forming circRNAs, in the process of lariat-driven circularization^26^. Two intronic complementary sequences (ICSs) pair with complementary non-adjacent introns, thus forming a loop containing both introns and exons. Splicing removes introns on the loop, thus forming circRNAs, in intron-paring-driven circularization^26^. RBP connects non-adjacent introns, so that the splice donor and the splice acceptor close and form a circle and then are spliced, forming circRNAs, in cyclization through RBP^27^. Researchers have found that the RBP Muscleblind binds flanking introns of circMbl, thereby promoting the biogenesis of circRNA^31^. Moreover, Liang et al.^32^ have found that after perturbation of the canonical pre-mRNA processing event, circRNA production tends to be affected.

The regulatory mechanism of circRNAs is associated with their generation mechanisms. Guarnerio et al.^33^ have found that in acute myeloid leukemia, chromosomal translocation causes 2 ICSs to be transferred to the same molecule and form paired ICSs flanking the translocation breakpoint, thereby promoting the production of circRNAs. Studies have found that in organisms producing circRNAs, the occurrence is mainly mediated by epithelial-mesenchymal transition (EMT)^34^. The dimeric protein Quaking, the main regulator of this process, can interact with the recognition element on the flanking intron, thus bringing the looped exons on the mRNA close to each other and promoting the production of circRNAs^27^. These are the possible mechanisms through which circRNAs are upregulated in tumors. Bachmayr-Heyda et al.^35^ have found that circRNAs can be degraded by miRNAs when they are completely paired with miRNAs; furthermore, the concentration of circRNAs in rapidly proliferating cells is lower than that in normal cells, possibly because of the dilution of circRNAs in vigorously dividing cells. These are the possible mechanisms through which circRNAs are down-regulated in tumors.

## Functions of circRNAs

In terms of biological function, circRNAs mainly act as miRNA sponges, gene transcription and expression regulators, and RBP sponges, circRNAs can also be translated into peptides.

Many circRNAs have one or more miRNA complementary binding sites, which, through complementary base pairing, bind and inhibit the function of miRNA; that is, they decrease the negative regulation of a target gene by miRNA^36^. This mechanism is currently considered the main function of circRNAs in tumors. Chen et al.^37^ have identified that circPVT1 binging to miRNA-125 family members up-regulates the expression of E2F2, thereby promoting the proliferation of gastric cancer cells. Zhang et al.^38^ have found that circUBAP2 up-regulates anti-apoptotic Bcl-2 protein expression and inhibits apoptosis of osteosarcoma cells. Furthermore, circNSD2 is highly expressed in human colorectal cancer tissues and cell lines, and acts as an miR-100b-5p sponge, thus targeting the downstream genes DDR1 and JAG1, and promoting the invasion and migration of colorectal cancer tumors^39^. The above evidence shows that circRNAs are closely associated with the regulation of tumor proliferation, apoptosis, and invasion.

Some circRNAs have one or more RBP binding sites, which can function as RBP sponges. Du et al.^40^ have found that circ-Foxo3 forms a circ-Foxo3-p21-CDK2 ternary complex with p21 proteins and cyclin-dependent kinases 2 (CDK2). This complex achieves the tumor-suppressing effect by inhibiting the function of CDK2 and preventing the cell cycle from transitioning from the G1 phase to the G1/S phase, thus having a tumor-suppressing effect. Yu et al.^41^ have found that circBIRC6 in the Ago2 complex of RBP increases in concentration, and directly binding with miR-34a and miR-145, thereby regulating and maintaining the pluripotency of human embryonic stem cells and inhibiting human embryonic stem cell differentiation. The presence of p53 binding sites on CircFoxo3 promotes the ubiquitination of p53 induced by MDM2, thus leading to the overall degradation of p53 protein^42^.

Some circRNAs also regulate gene transcription in cis or trans. Li et al.^25^ have found that EIciRNA, which is mainly located in the nucleus, interacts with U1 small nuclear ribonucleic protein (U1 snRNP) and promotes the transcription of the parental genes of EIciRNA. In addition, during the formation of circMbl, circular splicing of exons can compete with linear splicing, thus affecting the formation of linear RNA and consequently the expression of genes^31^.

Because circRNA does not have 3′ and 5′ ends, and lacks an internal ribosome entry site, it was originally thought to be unable to encode polypeptide chains. However, studies have since shown that the insertion of ribosome entry sites into circRNAs can result in binding to the 40S subunit of eukaryotes and subsequent translation^43^. For example, circZNF609 has an open reading frame, which can be translated into the protein FBXW7-185aa during myogenesis in a splicing-dependent manner, thus providing an example of circRNA-encoded proteins in eukaryotes^44^.

## CircRNAs associated with BC

With the development of high-throughput sequencing technology, circRNAs have been found in BC tissues and have been determined to be expressed differentially in normal breast tissues and BC tissues^45^. Therefore, identifying differentially expressed circRNAs may aid in the identification of novel and stable candidate biomarkers for the diagnosis and treatment of BC (**Figure 1**). **Table 1** shows the potential functional circRNAs associated with BC.

**Table 1:** The potential functional circRNAs in breast cancer

circRNAs	Associated miRNAs	Function	Reference
Upregulated			
circVAPA	miR-130a-5p	Oncogene	^46^
has_circ_0125597	miR-660	Oncogene	^105^
circACAP2	miR-29a/b-3p	Oncogene	^106^
has_circ_0061825	miR-326	Oncogene	^47^
has_circ_002178	miR-328-3p	Oncogene	^48^
has_circ_0069094		Biomarker	^16^
has_circ_0079876		Biomarker	
has_circ_0007255	miR-335-5p	Oncogene/biomarker	^49^
has_circ_0002453	miR-208a miR-3164	Oncogene/biomarker	^107^
has_circ_0000519	miR-703-3p	Biomarker	^108^
has_circ_100219	miR-485-3p	Oncogene	^50^
circAGFG1	miR-195-5p	Oncogene	^109^
has_circ_0072309	miR-492	Anti-oncogene/biomarker	^54^
has_circ_001569	miR-145	Oncogene	^57^
has_circ_0067934	miR-320	Oncogene	^110^
circKIF4A	miR-375	Oncogene/biomarker	^111^
circDENND4C	miR-200b/c	Oncogene	^112^
circRNACER	miR-136	Oncogene/biomarker	^113^
has_circ_001783	miR-200-3p	Oncogene	^114^
has_circ_21439	miR-26b miR-335	Biomarker	^115^
has_circ_0006528	miR-7-5p	Oncogene	^58^
has_circ_0103552	miR-1236	Oncogene	^72^
CircUBE2D2	miR-1236 miR-1287	Oncogene	^73^
hsa_circ_0000479	miR-4753/6809	Oncogene	^116^
hsa_circ_0001846	miR-661	Oncogene	^117^
hsa_circ_0005230	miR-618	Oncogene	^118^
hsa_circ_0005505	miR-3607	Oncogene	^59^
hsa_circ_0007294	miR-148a-3p miR-152-3p	Oncogene	^119^
hsa_circ_0007534	miR-593	Oncogene	^120^
hsa_circ_0008039	miR-432-5p	Oncogene	^121^
hsa_circ_0052112	miR-125a-5p	Oncogene	^122^
hsa_circ_0072995	miR-30c-2-3p	Oncogene	^123^
Downregulated			
has_circ_0017650		Biomarker	^16^
has_circ_0017536		Biomarker	
has_circ_0087378	miR-1260b	Biomarker	^124^
has_circ_0001451	miR-197-3p	Anti-oncogene/biomarker	^125^
circAHNAK1	miR-421	Anti-oncogene	^126^
circITCH	miR-214 miR-17	Anti-oncogene/biomarker	^127^
has_circ_11783	miR-365-5p	Biomarker	^115^
circASS1	miR-4443	Anti-oncogene	^51^
has_circ_0025202	miR-182-5p	Anti-oncogene	^87^
CircKDM4C	miR-548p	Anti-oncogene	^85^
hsa_circ_0000911	miR-449a	Anti-oncogene	^52^
hsa_circ_0001098	miR-3942	Anti-oncogene	^53^
hsa_circ_0007874	N/A	Anti-oncogene	^128^

### Progression and metastasis associated circRNAs in BC

Li et al.^16^ have investigated the profiles of differentially expressed circRNAs on the basis of competition in an endogenous RNAs (ceRNA) microarray, to construct a genome-wide circRNA profile. In that study, the authors detected a total of 4,370 differentially expressed circRNAs. Gene Ontology (GO) analysis and Kyoto Encyclopedia of Gene and Genome (KEGG) pathway analysis showed that these circRNAs were significantly associated with the cell cycle, DNA replication, and BC. Among them, 2,375 circRNAs were up-regulated, and 1,995 were down-regulated in cancerous tissues compared with adjacent normal tissues. Li et al. have verified the 4 most significantly up-regulated and 4 most down-regulated circRNAs, among which hsa_circ_0069094, hsa_circ_0079876, hsa_circ_0017650, and hsa_circ_0017536 show significantly different expression levels in plasma from patients with BC and normal human plasma. Generally, among the abnormally expressed circRNAs verified in BC, up-regulated circRNAs are often demonstrated to be carcinogenic, and down-regulated circRNAs are often demonstrated to be anti-carcinogenic. For example, circVAPA is up-regulated in BC tissues and cells, and it promotes BC migration, invasion, and proliferation through sponging miR-130a-5p, but it cannot regulate its parental genes^46^. Pan et al.^47^ have performed *in vitro* and *in vivo* experiments to evaluate the function of hsa_circ_0061825 (circ-TFF1) in the biological processes of BC cells. hsa_circ_0061825 is up-regulated in BC. Circ-TFF1 acts as a ceRNA of TFF1 by sponging miR-326. Furthermore, knockdown of circ-TFF1 hinders BC cell proliferation, migration, invasion, and EMT *in vitro* and controls tumor growth *in vivo*. More validated up-regulated circRNAs regulatory axes include hsa_circ_002178/miR-328-3p/COL1A1^48^, hsa-circ_0007255/miR-335-5p/SIX2^49^, and hsa_circ_100219/miR-485-3p/NTRK3^50^. Hou et al.^51^ have found that the circular RNA circASS1 is down-regulated in BC. The circRNA binds miR-4443 as a tumor promoter gene in the BC cell line MDA-MB-231 and inhibits the invasion and migration of BC cells. Similarly, the down-regulated circRNA regulatory axes include hsa_circ_000911/miR-449a/Notch1^52^ and Hsa_circ_0001098/miR-3942/BARD1^53^. In contrast, a recent study by Yan et al.^54^ has shown that hsa_circ_0072309 is upregulated in BC tissues compared with adjacent normal tissues, but Hsa_circ_0072309 inhibits the proliferation, migration, and invasion of BC cells by inhibiting miR-492. That study has provided the first evidence that the hsa_circ_0072309-miR-492 axis plays a vital role in BC progression.

In addition to acting as miRNA sponges, some circRNAs function through direct or indirect interactions with cancer-related signaling pathways. Phosphoinositide 3-kinase (PI3K)/protein kinase (AKT) signaling plays an important role in a variety of cancer behaviors^55^. The PI3K/AKT pathway acts as a key regulator of many important cellular processes and is the main signaling cascade activated in a variety of human cancers^55,56^. Xu et al.^57^ have found that the overexpression of hsa_circ_001569 in BC tissues and cell lines may promote the activation of PI3K-AKT signaling in BC cells, which is associated with lymph node metastasis, clinical stage, and poor prognosis. Western blot results have shown that knocking down hsa_circ_001569 decreases the levels of p-PI3K and p-AKT proteins, thus indicating that hsa_circ_001569 may act as a tumor promoter in BC by regulating the PI3K/AKT pathway and may promote the progression of BC. Multivariate analysis has confirmed that hsa_circ_001569 can be used as an independent prognostic factor for 5-year overall survival.

Both the invasion and migration of BC cells contribute to BC metastasis. Studies have shown that circRNAs not only participate in the carcinogenesis of BC but also play important roles in the metastasis of BC. The MAPK/ERK signaling pathway has important regulatory roles in cell proliferation, apoptosis, and metastasis, whereas Raf1 activates MAPK/ERK kinase (MEK) 1/2 dual-specificity protein kinases and then activates MAPK. Gao et al.^58^ have found that the up-regulation of circ_0006528 significantly increases the phosphorylation levels of MEK1/2 and ERK1/2 in MDA-MB-231 cells, and have verified that hsa_circ_0006528 activates the MAPK/ERK pathway *via* the hsa_circ_0006528/miR-7-5p/Raf1 axis, thereby promoting the metastasis of BC. Wu et al.^59^ have found that circIRAK3 expression increases in metastatic BC cells and is predictive of BC recurrence. Through the circIRAK3/miR-3607/FOXC1 signal cycle, circIRAK3 plays a regulatory role in BC metastasis. Knockout of circ_IRAK3 significantly inhibits BC lung metastasis *in vivo*, whereas FOXC1 expression is diminished in BC lung metastases.

Studies have confirmed that circRNA biogenesis is mainly mediated by EMT^34^, and EMT can temporarily cause loss of the epithelial characteristics of tumor cells, which acquire mesenchymal traits and no longer have apicobasal polarity and tight junctions, and may subsequently promote cell progression and metastasis^60^. Some researchers believe that exosomes may play roles in the progression and metastasis of BC. The level of exosomes in the serum is higher in patients with BC than in normal control patients^61^. Exosomes that are secreted by cancer cells and cancer-associated fibroblasts and released into the extracellular can transport the contents [non-coding RNA (ncRNA), mRNA, proteins, and other biologically active substances] to nearby or distant cells through the blood^62^. For example, Zhang et al.^63^ have identified the lncRNA MALAT1 in serum exosomes from patients with BC, and have determined that this lncRNA plays a carcinogenic role by activating WNT-β-catenin signaling. Therefore, the role of exosomes in the progression and metastasis of BC is worthy of further exploration.

### Diagnosis and prognosis associated circRNAs in BC

Previous studies have confirmed that circRNAs are closely associated with a variety of cancers, and have characteristics such as diversity^64^, stability^65^, specificity^28^, and high expression^26^, thus showing the potential of circRNAs as biomarkers for BC diagnosis and prognosis. The key to successful treatment of BC depends largely on the tumor stage at the time of BC diagnosis: the 5-year survival rate is >90% for early stage diagnosis but 20% for diagnosis after distant metastasis^66^. Methods currently used for diagnosing BC include CT, MRI, and histopathology. The disadvantages of these techniques are expense or invasiveness. Therefore, finding less costly methods that can aid in early diagnosis is important. A study by Yin et al.^67^ has screened for the expression profiles of plasma circRNAs from 57 patients with BC and 17 age-matched healthy individuals. A total of 41 abnormally expressed circRNAs have been identified through circRNA microarray analysis. The authors have verified by RT-PCR that hsa_circ_0001785, hsa_circ_0108942, and hsa_circ_0068033 were differentially abundant. After expanding the study cohort (*n* = 20), through ROC curve analysis, the authors found that hsa_circ_0001785 has the best diagnostic value among these candidates (AUC = 0.771). Currently, CA15-3 is the most widely used peripheral blood biomarker for BC detection^68,69^. CEA is used as a wide range of tumor biomarkers to monitor recurrence in postoperative cancer patients^70^ but is less sensitive in tumor detection. Yin et al.^67^, through ROC curve analysis, have found that, compared with CA15-3 (0.629) and CEA (0.562), hsa_circ_0001785 has a higher AUC value (0.784) and significantly higher sensitivity in tumor detection. Together, these findings suggest the diagnostic value of hsa_circ_0001785 for BC.

A study by Li et al.^71^ has used qPCR to measure circ-VRK1 expression in 350 BC tissues and 163 BC adjacent tissues; the results indicated that circ-VRK1 is downregulated in BC tissues, is associated with lower tumor stage and better survival, and can be used as an independent predictor of better overall survival. Yang et al.^72^ have found that the high expression of has_circ_0103552 in BC tissues and cells is associated with tumor clinical severity and poor prognosis. This circRNA functions as an miR-1236 sponge. Wang et al.^73^ have screened and identified a new circRNA, circ-UBE2D2, which is highly expressed in BC cell lines and tissues and can serve as a molecular sponge for miR-1236 and miR-1287, thus regulating the expression of their respective target genes. Subsequently, the proliferation, migration, and invasion of BC cells are promoted, a response associated with the clinical characteristics of tumor aggressiveness and poor prognosis. The above circRNAs can be used as prognostic biomarkers of BC.

The previous examples show the potential of circRNA to participate in the diagnosis and prognosis of BC. However, further clinical studies are needed before circRNAs can be recommended for diagnosis of human BC. With the continuous improvement of quantitative detection methods, such as RNA-seq, circRNAs are increasingly being discovered and are expected to become indicators for the diagnosis and assessment of BC subtypes. Moreover, the development of a circRNA scoring system is expected to provide guidance for clinical diagnosis and risk stratification of BC.

In previous studies, most circRNAs have been detected in tissues and cell lines, and some studies have suggested that circRNAs can be detected in peripheral blood exosomes. More than 58,330 circRNAs, 15,501 lncRNAs, and 18,333 mRNAs have been verified to be expressed in exosomes^74^. Exosomes are nano-sized membrane-bound vesicles that enclose various types of biologically active substances, including mRNAs and ncRNAs^75^. Exosomes can be isolated from the blood through a variety of methods, including binding, immunoaffinity capture, and differential ultracentrifugation, and can be used as biomarkers for the diagnosis and monitoring of tumor progression^76^. Furthermore, researchers have found that tumor cells secrete approximately 10 times more exosomes than normal cells^77^. The number of circRNAs verified in human serum exosomes exceeds 1,000, including ciRS133^78^ identified in serum from patients with gastric cancer and circIARS^79^ identified in serum from patients with pancreatic cancer. circRNAs can enter the blood circulation and can be easily measured in the serum^80^. However, 12,316 lncRNAs and 17,102 mRNAs have been identified in BC exosomes, but circRNAs have not been identified (www.ExoRBase.org)^74^. Therefore, more research must be conducted to confirm whether exosome circRNAs are present in the serum in patients with BC and further explore their possibility as diagnostic biomarkers.

### Chemoresistance associated circRNAs in BC

Chemotherapy is an effective strategy for the clinical treatment of patients with BC. Chemotherapy drugs include doxorubicin, paclitaxel, 5-fluorouracil (5-FU), and cyclophosphamide^81^. Most patients initially respond well to chemotherapy drugs, but as treatment progresses, the tumor evolves into a more invasive type, becomes resistant to conventional chemotherapy treatments, and eventually relapses, thus posing obstacles in BC treatment^82^. Resistance to BC treatment is regulated through a variety of mechanisms. CircRNAs mainly act as miRNA sponges, thus affecting the interaction of anti-tumor drugs with targets, influencing apoptosis-related pathways, and regulating gene expression at the transcriptional or post-transcriptional level, thereby promoting or reversing BC chemical resistance.

Yang et al.^83^ have determined the expression of CDR1-as and miR-7 in 5-FU chemosensitivity in BC cells by RT-PCR, and have found that in 5-FU resistant BC cells, CDR1as increases, and miR-7 is suppressed. miR-7 inhibits the target gene CCNE1 and promotes the chemical sensitivity of BC tissues to 5-Fu; therefore, CDR1as may regulate the chemosensitivity of 5-FU-resistant BC cells by inhibiting miR-7 and regulating CCNE1. Doxorubicin is the most commonly used anthracycline chemotherapeutic drug and the first-line drug for BC treatment. It embeds in the DNA base fragments of cancer cells, thus hindering the transcription and replication of DNA, and inhibiting tumor growth^84^. Liang et al.^85^ have demonstrated that circKDM4C significantly inhibits BC proliferation, metastasis, and doxorubicin resistance *in vitro* and *in vivo*, thus suggesting that the treatment targeting of the circKDM4C/miR-548p/PBLD axis may be a desirable treatment target of patients with BC. Tamoxifen is the most commonly used endocrine treatment for patients with hormone receptor-positive BC; as a selective estrogen receptor modulator, it competes with estradiol and decreases the activity of cancer cells^86^. However, many patients develop resistance after treatment. Sang et al.^87^ have established a tamoxifen-resistant MCF-7 cell line for research and confirmed that hsa_circ_0025202 acts as a miRNA sponge for miR-182-5p and further regulates the expression and activity of FOXO3a. Through miR-182-5p, the FOXO3a axis achieves tumor suppression and tamoxifen sensitization by regulating miR-182-5p. Uhr et al.^88^ have found that hsa-miR-7 is regulated by circRNA CDR1-AS, whereas hsa-miR-7 is associated with the prognosis of BC, and its high expression can predict adverse reactions to tamoxifen therapy. Taxanes act on the microtubule system, promote the polymerization of tubulin, and inhibit the disaggregation of tubulin, thus forming stable non-functional microtubule bundles and blocking the cell cycle in M phase, thereby preventing tumor cells mitosis and achieving anti-tumor effects. Chemical resistance of triple negative BC to paclitaxel has been one of the main problems in patients receiving chemotherapy. Ma et al.^89^ have found that circAMOTL1 promotes anti-apoptotic proteins and inhibits pro-apoptotic proteins by regulating the AKT pathway. It plays an important role in the paclitaxel resistance of BC cells. However, owing to insufficient research on the underlying mechanism and clinical research, whether circRNAs can provide new treatment strategies for future BC chemotherapy must be further verified.

## Research progress in circRNAs

Increasing advances in research technology are helpful for the preparation, detection, identification, and functional verification of circRNAs. Recently, the Cas9 homologous protein Csy4 in the CRISPR family has been found to cleave RNA molecules and maintain the stability of the 5′-end RNA product. On the basis of this characteristic, Borchardt et al.^90^ have designed a circRNA overexpression system. After insertion of the recognition sequence of Csy4 in the intron, Cys4 cuts the downstream competitive splicing signal of the sequence after co-expression and stabilizes the upstream cleavage product. This product forms a circRNA under the effect of the splicing signal, through technology exploiting the cyclization of RNA. Panda et al.^91^ have discovered and named a new method, RPAD [RNase R treatment followed by polyadenylation and poly (A) + RNA depletion]. Because circRNA is usually prepared by RNase R digestion to remove linear RNA, but residual linear RNA may interfere with the downstream analysis of circRNA, RPAD adds a step of removing poly (A), thus allowing many highly enriched circRNAs to be obtained. Hansen et al.^92^ have compared 5 bioinformatics algorithms (circRNA_finder, find_circ, CIRCexplorer, CIRI, and MapSplice) to identify circRNA. These common circRNA identification tools identify back-splicing sites in RNA-seq data. Related studies^7,93^ have shown that these tools generally have high-false positive and false-negative rates, and the coincidence rate of the same sequencing data detected with different tools is low, because the algorithm based on RNA-seq data uses only back-splicing site information to identify circRNAs but ignores the influence of other factors in the circularization process. A single algorithm always has limitations in some aspects, as a result of design problems; therefore, prediction of circRNA is recommended through use of 2 or more algorithms (**Table 2**). In recent years, machine learning methods have been increasingly used in bioinformatics research. Some studies^94,95^ have analyzed the factors influencing in the formation of circRNAs, trained traditional machine learning algorithms (such as support vector machine, random forest, and multi-core learning) to identify circRNAs, and achieved high recognition accuracy. Several tools based on sequence prediction of circRNAs, such as CirRNAPL^96^, DeepCirCode^97^, circDeep^98^, and CRC^99^, all mine local information from the sequence, but the upstream and downstream regulation of the sequence information, remote control information, RNA-protein interaction information, and RNA structure have not been discovered through existing tools. These aspects may serve as potential directions for the development of circRNA identification tools.

**Table 2:** circRNA identification software

Software	Feature	Website
Acfs^129^	Allows for de novo sequencing, and accurate identification and quantification of circRNAs from single- and double-ended RNA data	code.google.com/p/acfs
CIRCfinder^22^	Recognizes intron circular RNA	github.com/YangLab/CIRCfinder
circRNA_ finder^130^	Has high accuracy, independently of gene annotation information	github.com/orzechoj/circRNA_finder.git
CIRI-full^131^	Recognizes circRNA, assembles the complete nucleotide sequences, and quantifies alternative splicing products of circRNAs	sourceforge.net/projects/ciri-full
Circseq_cup^132^	Assembles full-length circRNA sequences on the basis of back-splicing RNA-seq and double-terminal RNA-seq data	ibi.zju.edu.cn/bioinplant
CircExplorer2^133^	Uses integrated multiple alignment algorithms to detect alternative splicing of circRNA, de novo synthesis, and assembly of full-length circRNA transcripts	github.com/YangLab/CIRCexplorer2
CIRI2^134^	Based on multiple sub-matching strategies and maximum likelihood estimation models; identifies back-splicing reads and filters false positives from repeated sequences and mapping errors	sourceforge.net/projects/ciri/files/CIRI2
CircView^135^	Reveals the structure of circRNA on the basis of species annotation information, allowing users to view the regulatory elements of circRNAs and predict potential functions	gb.whu.edu.cn/CircView
CircSplice^136^	Specific recognition of alternative splicing events within circRNA, enabling comparison of differences between groups of alternative splicing events of circRNAs	gb.whu.edu.cn/CircSplice
DCC^137^	Depends on filters and integrated data across repetitive sets to evaluate expression between circRNAs and host genes	github.com/dieterich-lab/DCC
find_circ^138^	Uses only the fasta sequence of the genome, independently of the gene annotation information	github.com/marvin-jens/find_circ
FUCHS^139^	On the basis of long read sequencing data, analyzes alternative splicing events in circRNAs, single and double breakpoint events, and the read coverage of circRNAs	github.com/dieterich-lab/FUCHS
KNIFE^140^	Relies on Bowtie2 for multi-stage correction, combined with read mapping quality and correction quality for static modeling detection of circRNAs	github.com/lindaszabo/KNIFE
MapSplice^141^	Tag alignment and splice inference (two-step method)	www.netlab.uky.edu/p/bioinfo/MapSplice
PRAPI^142^	Vector drawing of circRNAs	www.bioinfor.org/bioinfor/tool/PRAPI
segemehl^143^	Recognizes circRNAs and detects splicing, trans-splicing, and gene fusion events	www.bioinf.uni-leipzig.de/Software/segemehl
UROBORUS^144^	On the basis of total RNA sequencing data, accurately predicts circRNAs with low expression, but cannot predict circRNAs and circRNAs formed by gene spacers	uroborus.openbioinformatics.org

Currently, the circRNA identification methods commonly used in laboratories include the RT-PCR quantitative method^100^, probe hybridization method^101^, and two-dimensional denaturing polyacrylamide gel (2D-PAGE) electrophoresis^102^. Multiple methods are often used in research to identify circRNAs. For example, Ghorbani et al.^103^ have used multiple methods, such as RT-PCR, RNase R, 2D-PAGE, and northern blotting, to identify circRNAs. The main methods for verifying the functions of circRNAs include constructing circRNA-deficient mutants, constructing circRNA overexpression vectors, and using RNA interference technology to silence or inhibit circRNA. However, these verification methods currently rely on the specific back-splicing of circRNA, and because the sequences of a single circRNA and its homologous linear RNA subtypes completely overlap, these methods must be further improved^104^.

## Conclusions and perspectives

This review summarizes the demonstrated links between circRNAs commonly found in BC and proliferation, metastasis, and chemotherapy resistance associated with BC cells. It further describes the potential of circRNAs as potential diagnostic and prognostic biomarkers, and therapeutic targets for BC in the future. CircRNAs are involved in a variety of pathological processes in BC. They have the characteristics of diversity, high expression abundance, stability, specificity, versatility, and conservation, and their clinical potential as biomarkers for BC has been demonstrated. However, many issues remain to be addressed, including the formation and regulatory mechanisms of circRNAs in BC, and whether the expression status of circRNAs in tumor tissues and serum exosomes is representative of BC status and can be used in BC diagnosis, prognosis, and treatment evaluation. Future research may combine multiple circRNAs to detect whether the sensitivity or specificity of diagnosis can be improved; detect circRNAs in peripheral blood or body fluids instead of tissue cells; and conduct long-term follow-up studies on large samples to explore the clinical value of circRNAs for diagnosis and treatment of BC.
